# *In vitro* 5-LOX inhibitory and antioxidant potential of isoxazole derivatives

**DOI:** 10.1371/journal.pone.0297398

**Published:** 2024-10-04

**Authors:** Waqas Alam, Haroon Khan, Muhammad Saeed Jan, Hany W. Darwish, Maria Daglia, Ahmed A. Elhenawy

**Affiliations:** 1 Department of Pharmacy, Abdul Wali Khan University Mardan, Mardan, Pakistan; 2 Department of Pharmacy, Bacha Khan University, KP, Charsadda, Pakistan; 3 Department of Pharmaceutical Chemistry, College of Pharmacy, King Saud University, Riyadh, Kingdom of Saudi Arabia; 4 Department of Pharmacy, University of Napoli Federico II, Naples, Italy; 5 International Research Center for Food Nutrition and Safety, Jiangsu University, Zhenjiang, China; 6 Chemistry Department, Faculty of Science, Al-Azhar University, Nasr City, Cairo, Egypt; Vignan Pharmacy College, INDIA

## Abstract

5-Lipoxygenase (5-LOX) is a key enzyme involved in the biosynthesis of pro-inflammatory leukotrienes, leading to asthma. Developing potent 5-LOX inhibitors are highly attractive. In this research the previously synthesized isoxazole derivatives has been investigated against 5-LOX inhibitory and antioxidant in vitro assay. The compound 3 caused concentration dependent inhibition of 5-LOX with overall IC_50_ value of 8.47 μM. The investigated compounds **C5** also exhibited good 5-LOX inhibitory effect. The IC_50_ demonstrated for **C5** was 10.48. Among the 10 synthesized compounds, the potential 5-LOX inhibitory effect was reported for **C6**. The most potent compound which showed excellent free radical scavenging effect was **C3** having IC_50_ value of 10.96 μM. The next most potent antioxidant activity was reported for **C5** which non-significantly showed free radical scavenging effect. The IC_50_ value observed for **C5** was 13.12 μM. Compound **C6** also showed potent dose dependent antioxidant effect with IC_50_ value of 18.87 μM having percent inhibition of 91.63±0.55, 88.45±0.49, 83.53±0.45, 78.42±0.66 and 73.72±0.64 at concentration 1000–62.5 μg/mL respectively. Among the tested compounds, **C6** was found most potent which showed significant 5-LOX percent inhibition assay and also reported the minimum IC_50_ value comparable to the reference drug. The *in vitro* 5-LOX enzymes inhibition assays of **C5** and **C3** also showed excellent percent inhibition and good potency next to **C6**. We concluded that amongst the investigated designed molecules the **C3** was found best potent and showed significant dose dependent antioxidant activity against DPPH screening. The IC_50_ value reported for **C3** was found good as compared to standard drug. Moreover, **C5** and **C6** also showed excellent free radical scavenging effect against DPPH assay. Computational methods have also been employed to explore the probable interaction model of inhibitors and enzyme active sites, and also to correlate the results of *in silico* and *in vitro* studies.

## 1 Introduction

The Leukotriene (LTs) are the oxygenated products of lipoxygenase (LOX) cascades notorious for induction of inflammatory reactions [1]. The oxygenated products of 5-Lipooxygenase (5-LOX) are the key eicosanoids [2] like LTB4 (leukotriene B4) which accelerate several inflammatory and allergic conditions [3]. LOX enzymes are discovered in many plants and animals, but not in bacteria or yeast. The enzyme 5-lipoxygenase (5-LOX) is responsible for the biosynthesis of leukotrienes from the precursor AA [4]. Fatty acids are oxidized by non-heme iron enzymes like LOX to produce lipid hydroperoxides [5]. The presence of 5-LOX and its interaction with arachidonic acid is a vital first phase in the production of leukotrienes (LTs). In typical cellular structures, the release of arachidonic acid (AA) is primarily facilitated by endogenous phospholipids. This process is thought to be mediated by cytosolic phospholipases, which work in conjunction with many other enzymes, notably LT-converting phospholipase A2. Lipid peroxides are mostly formed as a result of LOXs [6].

Previously the Abibindhu *et al* synthesized various isoxazole derivatives and explored for both COX-2 and 5-LOX inhibitory potential [7]. Sadiq *et al*., tailoring the substitution pattern of Pyrrolidine-2, 5-dione and explored it for dual COX-2/5-LOX inhibition [8]. Alsheri explored the various fraction and isolated compounds form *Habenaria plantegania* Lindl. for dual COX/LOX inhibition *Habenaria plantegania* Lindl [9].

The pathophysiological role of 5-LOX in respiratory and cardiovascular diseases has been revealed by subsequent studies that have examined the enzymatic pathway of 5-LOX. 5-LOX is a non-heme metalloenzyme that has iron at its active site; iron is present in ferrous form during rest and in ferric form during active state; hydroperoxidation of lipids due to physiological stress may activate 5-LOX; it has been demonstrated that the activated enzyme in the cytoplasm is translocated to the nuclear membrane upon Ca^+2^ binding and/or by phosphorylation [10]. Leukotrienes are known to be lipid mediators for inflammatory response with major roles in cardio vascular diseases [11]. Results from recent studies also suggest the role of leukotrienes in prostate cancer, osteoporosis and in certain forms of leukemia. 5-LOX is validated as a potential drug target for treating asthma, rheumatoid arthritis, osteoarthritis, allergic, cardiovascular diseases and certain types of cancer. For more than three decades considerable progress has been made towards finding more potent 5-LOX inhibitors. Despite considerable efforts made towards the development of efficient drugs that target 5-LOX enzyme, zileuton remains the only clinically approved 5-LOX inhibitor in the treatment of asthma but its use is limited due to severe side effects such as, weak potency, liver toxicity, and an unfavorable pharmacokinetic profile with a short half-life [12, 13].

Isoxazole compounds have a broad range of pharmacological actions and targets. One trend has been creating chemicals with heterocycle rings. Enhancement of physical-chemical characteristics can be provided by integrating the isoxazole ring. The isoxazole ring is becoming a common component in compound development because to its distinctive features [14]. The antioxidant potential of organic compounds must be evaluated as they are used in food, medicine, and cosmetics [15]. Reactive species are produced in living systems by a variety of metabolic processes and stressful circumstances. They are primarily reactive oxygen species (ROS). Raised levels of ROS may alter biomolecules’ activities and impair their structural integrity, which can cause cellular malfunction and even premature death of cells. A rise in ROS over time can lead to oxidative stress at the systemic level, which manifests as a number of health issues including cancer, inflammation, age-related disorders, and heart problems [16, 17]. The most common and simple colorimetric technique for assessing the antioxidant capabilities of pure molecules is the DPPH assay, which is frequently used to determine how well a certain antioxidant molecule scavenges free radicals [18].

Researchers have reported antioxidant potential of isoxazole derivatives. It was found that the compounds’ varying potencies were mostly dependent on the electronic characteristics of the substituents on the phenyl ring of isoxazole, namely their ability to withdraw and release electrons [19]. Palleapat et al., conducted the MABA assay to screen for antitubercular activity against Mycobacterium TB, with isoniazid serving as a positive control to compare the results. According to the investigations, isoxazole-1-carboxamides exhibited more activity in contrast to their comparable chalcone counterparts [20]. Bhatia *et al*., has reported the antioxidant effect of indole-functionalized isoxazoles derivatives. They found that in the DPPH test, the created compounds showed variable capacity to scavenge free radicals. The isoxazole ring of this molecule is considered as potent antioxidant moiety. The compounds’ capability to inhibit free radicals was significantly influenced by the substituted sequence at the phenyl ring connected to the isoxazole group [21]. Therefore, there is a strong need for the development of safer and more potent 5-LOX inhibitors. In the present study we have investigated the already synthesized ten pyrimidine isoxazole derivatives and evaluated their 5-LOX inhibition and antioxidant *in vitro* activities.

## 2 Materials and methods

### 2.1. Chemicals

Leukotriene (CAS 71160-24-2, Icatibant (CAS 138614-30-9). DPPH, Montelukast (Montiget by Getz pharma).

### 2.2. Synthesis

A series of isoxazole derivatives were previously synthesized and characterization has been done [22].

### 2.3. 5-LOX inhibitory assay

The synthesized Isoxazole derivatives will be investigated for 5-LOX Inhibition studies as per established protocols of Wisastra and his co researchers [23]. In this experiment, residual enzyme potential will be utilized to measure the degree of enzyme inhibition after 10 to 15 minutes of inhibitor’s incubation at 25°C. The reaction involving the conversion of linoleic acid, a substrate of lipoxygenase, into hydroperoxy-octadecadienoate (HPOD) will be employed as a means of quantifying the said reaction. The variation in the rate of absorption will be determined by employing a UV-visible spectrophotometer set at a wavelength of 234 nm. A 50 mM Tris buffer containing 2 mM EDTA and 2 mM CaCl_2_ was used as the assay buffer in this experiment. The buffer has a pH of 7.5. Following this, a buffer solution was employed to dilute the 5-lipoxygenase (5-LOX) enzyme at a concentration of 20,000 units per millilitre (U/mL) at a ratio of 1:4000. The 100 mM inhibitor was diluted using the test buffer following the administration of dimethyl sulfoxide (DMSO) for its dissolution. The content of linoleic acid was reduced to 20 mg/ml through dilution using ethyl alcohol. To accomplish this, 1 mL of the enzyme solution (1: 4000) will be combined with 100 μL of 2 mM adenosine triphosphate (ATP), 100 μL of the inhibitor (1 mM), and 790 mL of Tris buffer while incubated for 10 min. Following approximately 10 seconds of the substrate and the enzyme mixing together, 10 μL of a 20 mM substrate solution will be added to the mixture. At that point, the changing rate of the substrate will be determined. The response rate will be utilized as a positive control without blockers. In this experiment, montelukast will be utilized as the reference medicine.

### 2.4. DPPH activity

The previously outlined methodology will be employed to evaluate the compounds’ antioxidant ability, as determined by the processes of the 1,1-diphenyl, 2-picrylhydrazyl (DPPH) free radical [24]. The experimental specimens will be subjected to dilution using different quantities, spanning from 62.5 to 1000 μg/ml, and afterwards introduced into a methanolic solution of DPPH at a concentration of 0.004%. At 517 nm, the absorbance will be determined by a UV spectrophotometer following a 30-minute duration. The scavenging capability of ascorbic acid will be quantified by employing the equation:

DPPH=[(A0−A1)/A0]100

Whereas:

A0: Absorbance value of the control

A1: Absorbance value of the investigated compound concentration.

Every test was carried out three times, and the median inhibitory dosages (IC_50_) were determined. The drawing of inhibition graphs was performed by GraphPad Prism software-GraphPAD, San Diego, California, USA.

### 2.5. Molecular docking studies

The docking experiment was performed using the co-crystallized inhibitors of each enzyme as follows: acarbose for ascorbate for peroxidase (PDB: 2X08) [25], and arachidonic acid for 5-lipoxygenase (PDB: 3v99) [26]. These inhibitors were selected based on their known binding affinities and structures. Using Glide’s module^®^ [27]. We performed the docking study for the target molecules and to employ it in the discovery studio. We obtained the crystals configurations of the initial inhibitors by installing them properly into their binding sites. We removed the H_2_O and inhibitors from the crystallized enzymes and added the H atoms. We redocked the examined ligands into the empty active sites after removing the standard inhibitors from it. To measure the binding affinity, the ChemPLP scoring function was applied, and the charges were determined by the CHARMM force field. The redocked poses of the inhibitors had RMSD values less than 2 Å compared to the original poses, which showed a reliable docking procedure. The structure with the lowest RMSD score was selected to generate different ligand poses in each case. The binding affinity was calculated using the ChemPLP scoring function, and the charges were assigned by the CHARMM force field.

### 2.6. Statistical analysis

The results of every investigation were presented as the mean SEM after being performed three times. A two-way ANOVA followed by Bonferroni posttest has been utilized to evaluate the positive control and investigated groups. *P* <0.05 has been used to predict the statistical significance.

### 2.7 IC_50_ calculation

The IC_50_ is the concentration of the antioxidant required to scavenge 50% of the initial DPPH radicals. The lower the IC_50_ value, the more effective the ingredient is in scavenging DPPH, implying higher antioxidant activity. The IC_50_ values were determined from dosage response curve using Microsoft Excel program.

## 3 Results and discussion

### 3.1 5-LOX inhibitory assay

**Fig 1:**
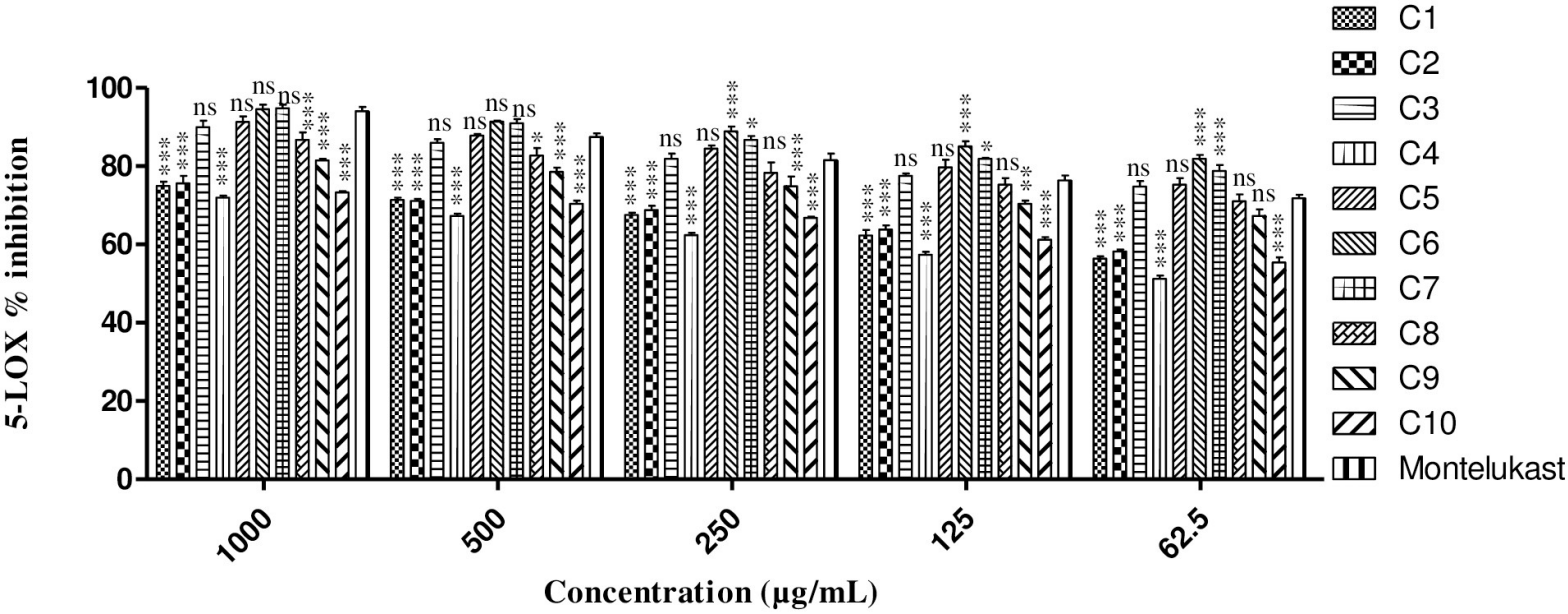
*In vitro* 5- LOX anti-inflammatory activity of synthesized isoxazole compounds against montelukast as standard. Data is illustrated as mean ± SEM (n = 3); significant differences are indicated by * = *P* < 0.05, ** = *P* < 0.01 and *** = P < 0.001, ns; not significant when compared to the positive control.

The *in vitro* result findings of 5-LOX inhibitory potential of the designed tested derivatives and their IC_50_ values are computed in Fig 1. The *in vitro* 5-LOX investigations of the **C1** exhibited significant percent inhibition of 74.94±1.07, 71.39±0.60, 67.58±0.56, 62.29±1.43 and 56.37±0.58 at different concentration of 1000–62.5μg/ml and the IC_50_ value determined for **C1** was 74.09 μM. Significant percent inhibition of 75.63±1.87, 71.12±0.54, 68.79±1.08, 63.79±1.88 and 58.20±0.47 were observed for **C2** at various concentrations with IC_50_ of 47.59 μM. The percent inhibition for **C3** were 89.93±1.73, 85.94±0.91, 81.90±1.32, 77.51±0.59 and 74.80±1.41 at various concentrations with IC_50_ of 8.47 μM. The percent inhibition of **C4** showed significant results having IC_50_ of 103.59 μM. The investigated compounds **C5** also exhibited good 5-LOX inhibitory effect. The non-significant percent inhibition for **C5** were demonstrated as 91.30±1.42, 87.78±0.45, 84.44±0.86, 79.72±1.89 and 75.29±1.64 at various concentration (1000–62.5μg/ml). The IC_50_ demonstrated for **C5** was 10.48. Among the 10 synthesized compounds, the potential 5-LOX inhibitory effect was reported for **C6**. The non-significant percent inhibition observed at concentration 1000 μg/ml and 500 μg/ml for **C6** were 94.58±1.12 and 91.40±0.20 respectively, whereas the significant percent inhibition values reported at concentration 250, 125 and 62.5μg/ml were 88.85±1.26, 85.08±0.47 and 81.90±0.96 respectively. The IC_50_ value found for **C6** was 3.67 μM. Similarly, the assayed compound **C7** also exhibited non-significant percent inhibition of 94.80±0.90 at 1000μg/ml and 90.94±1.13 at 500μg/ml whereas significant percent inhibition was reported as 86.72±1.01, 81.84±0.30 and 78.80±1.50 at 250, 125 and 62.5μg/ml, respectively. The percent inhibition for **C8** were established significant as 86.70±1.20 at 1000μg/ml and 82.31±1.20 at 500μg/ml whereas found non-significant as 78.30±2.67at 250μg/ml, 75.30±1.67 at 125μg/ml and 71.03±1.79 at 62.5 μg/ml. The IC_50_ calculations noted for **C7** and **C8** were 10.51 and 9.80 μM, respectively. The remaining investigated compounds i.e. Compounds **C9** and **C10** also Exhibited good activity against 5-LOX enzyme. The percent inhibition reported for **C9** were 81.42±0.43, 78.56±1.06, 74.90±2.45, 70.40±0.82, 67.33±1.66 and for **C10** were 73.31±0.35, 70.34±0.90, 66.78±0.34, 61.23±0.65, 55.34±1.34 respectively. The IC_50_ values determined for **C9** and **C10** were 11.25 and 67.06 μM, respectively. The percent inhibitions for montelukast as a standard drug was calculated as 94.08±1.04, 87.45±0.90, 81.58±1.63, 76.40±1.20 and 71.80±0.90 at 1000–62.5 μg/ml respectively. The IC_50_ values for montelukast was noted as 21.84 μM.

Lipoxygenases (LOXs) are non-heme iron-containing dioxygenases that generate hydroperoxy metabolites (HPETEs) by incorporating molecular oxygen into the substrate. There are six functioning LOX isoforms in humans [28]. The 5-lipoxygenase(5-LOX) is an isozyme of LOX that initiates the second crucial metabolic process that produces eicosanoids. They are quite common in mammals, fungi, and plants. Most myeloid cells that are associated with inflammatory and immunological responses, which include polymorphonuclear leukocytes (eosinophilsandneutrophils) and mononuclearcells (lymphocytes, monocytes, andmacrophages), express the 5-LOX protein [29, 30].

Leukotriene B4 is the final byproduct of the 5-LOX pathway and is a mediator of a number of inflammatory and allergic disorders, including atherosclerosis, cancer, and cardiovascular conditions. However, suppressing 5-LOX may aid to lessen the chance of cardiovascular and gastrointestinal problems triggered by COX-1/2inhibitors, respectively, by lowering leukotriene levels. Notable is the possibility that COX/5-LOX co-inhibition may lessen adverse effects on the cardiovascular and gastrointestinal systems while maintaining the main effect of COX-1/2 inhibitors [31, 32].

Rakesh et al., has reported both the COX/5-LOX *in vitro* inhibitory effect of 3,5-Disubstituted Isoxazole compounds. They found that the preference for the target sites is largely determined by the presence of the pharmacophores (3-methylthienyl) and (3,4,5-trimethoxypheyl) in the side chains of the isoxazole. The inclusion of aryl moiety with strong electron-donor substituents and moderately electron-rich heteroaryl substituents (indole) may be the cause of compounds considerable inhibitory activity [33].

As previously researchers have reported that both *in vitro* inhibition of COX/5-LOX has shown excellent anti-inflammatory effects. Hence, in this study the *in vitro* 5-LOX enzyme inhibition potential of each tested compound was evaluated. The effectiveness of the tested analogues has been calculated as IC_50_ in μM that is the amount of the compound that results in50%blockingof the enzyme. The result outcomes of our research established that the ten designed investigated isoxazole derivatives exhibited excellent 5-LOX blocking effect. The result findings of 5-LOX inhibition assays are represented in Fig 1. The 5-LOX enzymes inhibition analysis has been conducted for the tested derivatives at various concentration strengths. All the compounds showed significant percent inhibition of 5-LOX pathway in dose dependent manner. Among the tested compounds, **C6** was found most potent which showed significant 5- LOX percent inhibition assay and also reported the minimum IC_50_ value comparable to the reference drug. The *in vitro* 5-LOX enzymes inhibition assays of **C5** and **C3** also showed non- significant percent inhibition and good potency next to **C6**. The IC_50_ values of **C5** and **C3** were also found effective. The remaining compounds also showed significant percent inhibition against 5-LOX enzyme inhibition assay.

### 3.2 DPPH activity

**Fig 2:**
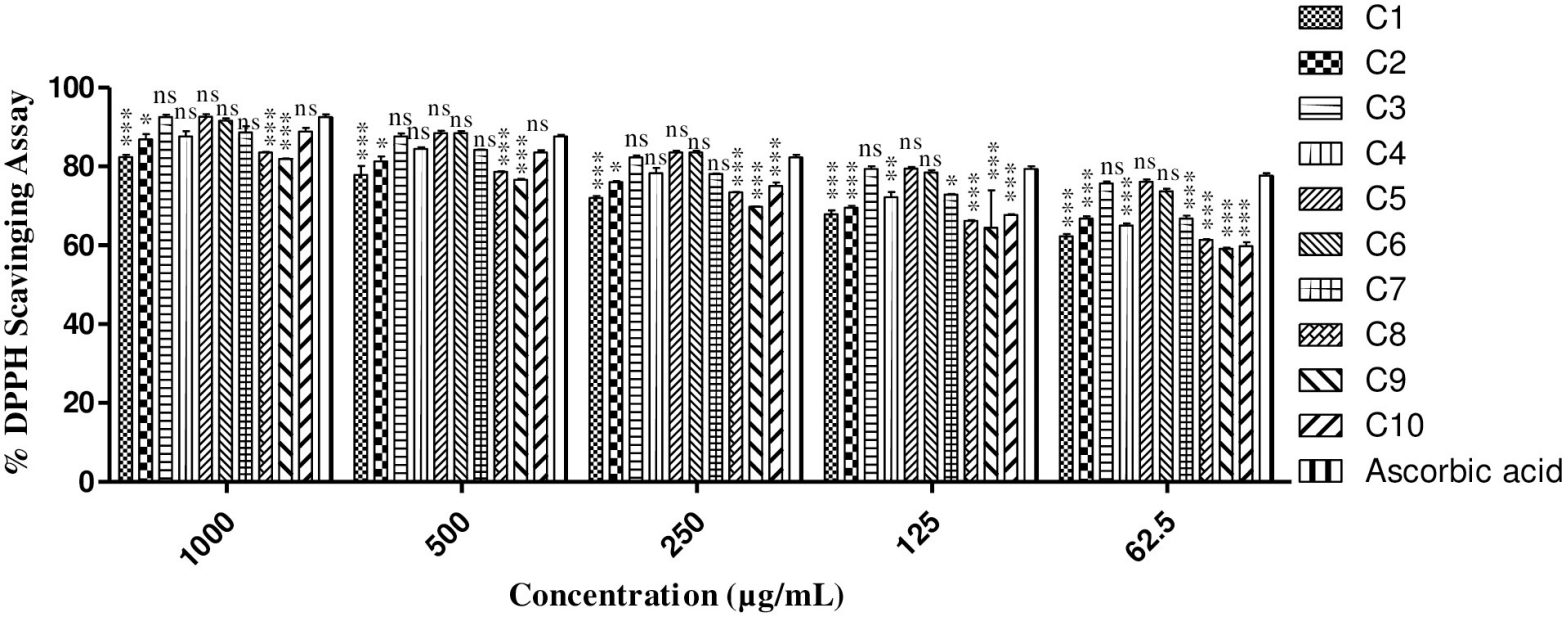
*In vitro* DPPH enzyme inhibition investigation of synthesized isoxazole derivatives, using ascorbic acid as reference drug. Data is illustrated as mean ± SEM (n = 3); significant differences are indicated by * = *P* < 0.05, ** = *P* < 0.01 and *** = *P* < 0.001, ns; not significant when compared to the positive control.

The *in vitro* antioxidant effect of the synthesized derivatives has been evaluated against DPPH enzymes activity. The result findings are represented in Fig 2 and showed that almost all compounds has excellent antioxidant effect against DPPH free radical scavenging activity. The most potent compound which showed excellent free radical scavenging effect was **C3** with different percent inhibitions of 92.51±0.62, 87.65±0.70, 82.25±0.55, 79.37±0.69 and 75.72±0.51 having IC_50_ value of 10.96 μM. The next most potent antioxidant activity was reported for **C5** which non- significantly showed free radical scavenging effect. The DPPH percent inhibition reported for **C5** was 92.63±0.64, 88.45±0.55, 83.53±0.41, 79.42±0.46 and 76.10±0.64 at different ranges of (1000, 500, 250, 125 and 62.5μg/mL) concentrations, respectively. The IC_50_ value observed for **C5** was 13.12μM. Compound **C6** also showed potent dose dependent antioxidant effect with IC_50_ value of 18.87 μM having percent inhibition of 91.63±0.55, 88.45±0.49, 83.53±0.45, 78.42±0.66 and 73.72±0.64 at concentration 1000–62.5 μg/mL respectively. The percent inhibition of **C1** and **C2** also expressed potent antioxidant effect against DPPH activity. At highest concentration (1000 μg/mL), the percent inhibition for **C1** and **C2** was 82.36±0.57 and 86.91±1.30 respectively. The reported IC_50_ value for **C1** and **C2** was 48.32 and 37.57 μM respectively. Compound **C4** also showed significant free radicle scavenging effect against DPPH activity. The percent inhibition established for **C4** at highest concentration (1000μg/mL) was 87.65±1.32 having IC_50_ value of 32.28 μM. The percent inhibition decreased to 65.03±0.48 at lowest concentration (62.5μg/mL). The present investigation of DPPH activity for **C7**, **C8**, **C9** and **C10** also showed that a significant excellent free radical scavenging effect was observed. The highest percent inhibition for **C7**, **C8**, **C9** and **C10** were 88.63±1.57, 83.53±0.20, 81.85±0.18 and 88.88±0.89 respectively at highest Concentration (1000μg/mL) assay which decreased to 66.78±0.72, 61.35±0.18, 59.12±0.34 and 59.82±0.95 respectively at lowest concentration (62.5 μg/mL). The IC_50_ values reported for **C7**, **C8**, **C9** and **C10** were 31.04, 54.71, 68.81 and 84.89 μM. Ascorbic acid, being used as a standard drug against DPPH antioxidant assay, demonstrated percent inhibition of 92.51±0.69, 87.65±0.42, 82.25±0.72, 79.37±0.71 and 77.72±0.59 at different concentration ranges of 1000–62.5 μg/mL respectively. The observed IC_50_ value for ascorbic acid was 17.90 μM.

The antioxidant potential of organic compounds must be evaluated as they are used in food, medicine, and cosmetics [15]. Reactive species are produced in living systems by a variety of metabolic processes and stressful circumstances. They are primarily reactive oxygen species (ROS). Raised levels of ROS may alter biomolecules’ activities and impair their structural integrity, which can cause cellular malfunction and even premature death of cells. A rise in ROS. Overtime can lead to oxidative stress at the systemic level, which manifests as a number of health issues including cancer, inflammation, age-related disorders, and heart problems [16, 17, 34]. The most common and simple colorimetric technique for assessing the antioxidant capabilities of pure molecules is the DPPH assay, which is frequently used to determine how well a certain antioxidant molecule scavenges free radicals [18]. Researchers have reported antioxidant potential of isoxazole derivatives. Bhatia *et al*., has reported the antioxidant effect of indole-functionalized isoxazole derivatives. They found that in the DPPH test, the created compounds showed variable capacity to scavenge free radicals. The molecule containing indole-functionalized isoxazole ring o is considered as potent antioxidant moiety. The compounds’ capability to inhibit free radicals was significantly influenced by the substituted sequence at the phenyl ring connected to the isoxazole group [21]. Yatoo and his co researchers reported the antioxidant potential of diosgenin based isoxazole derivatives. They found that the synthesized compounds exhibited highest antioxidant and anticancer effect [35].

**Fig 3:**
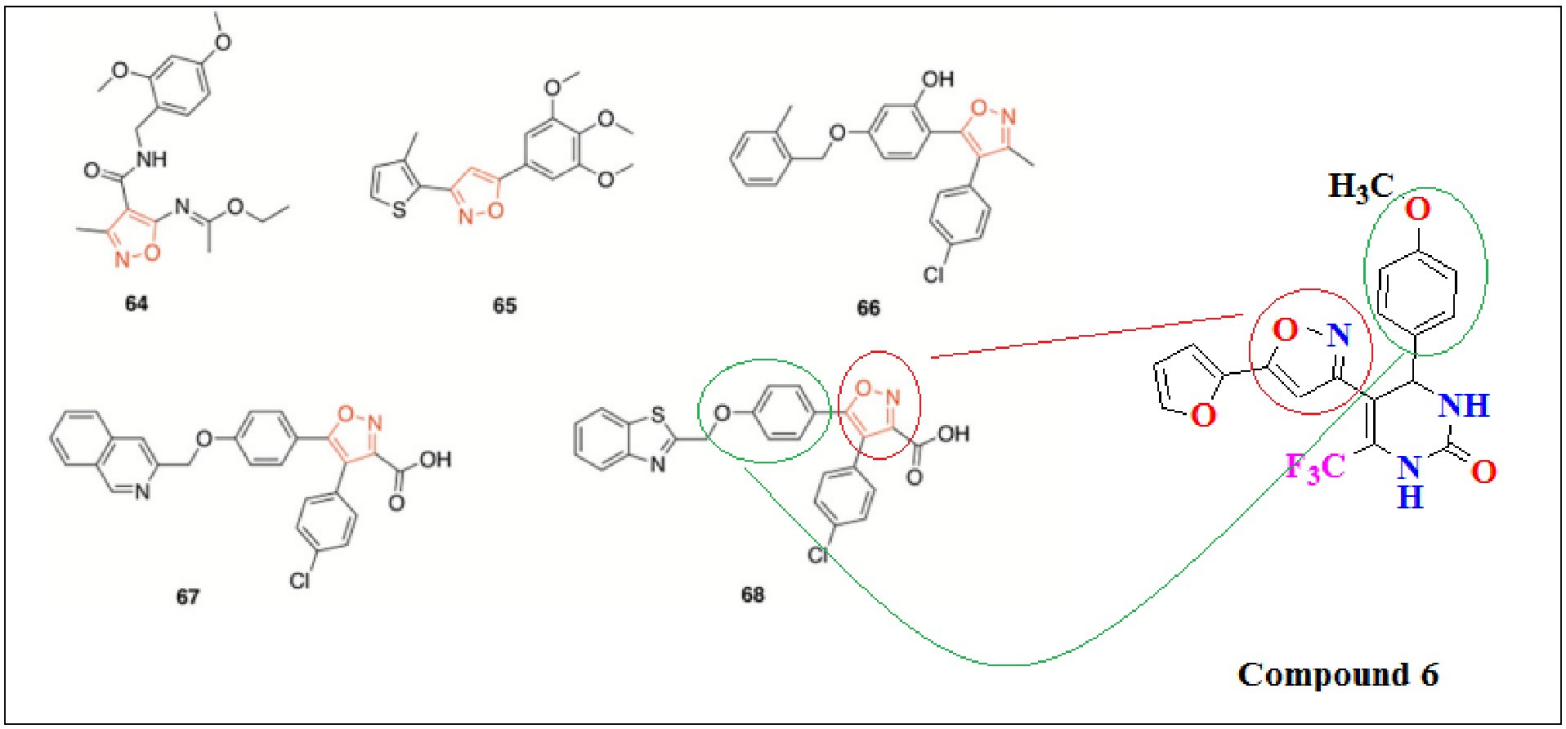
Structure similarity of previously isoxazole derivatives and current synthesized potent compound 6.

A novel series of isoxazole derivatives were synthesized and explored for LPS-induced TNF α production in human whole blood cell cultures. Among these, compound 64 was chosen as one of the most interesting. Compound 64 strongly inhibited the paw edema in a dose dependent manner. The results showed that it may be used as a medication to treat inflammation. LOX and COX are both are the key enzymes involved in inflammation (Fig 3) [14]. A series of isoxazole bearing derivatives were synthesized as screened for their anti-inflammatory activity via LOX and COX inhibition. Compound 65 considerably reduce the activity of COX and 5-LOX. in addition, it also displayed excellent inhibition on tumor growth in vivo and in vitro. This results result specified that compound 65 as a capable molecule against cancer and inflammation [33]. Based on the moiety of hit 66, which was earlier identified as a 5-LOX product synthesis inhibitor were designed, synthesized and pharmacologically evaluated. Two standard molecules 67 and 68 with extraordinary inhibitory potential against cellular 5-LOX product formation (IC_50_ = 0.24 μM, respectively) were disclosed (Fig 3) [36].

In this *in vitro* research, the antioxidant potential of synthesized tested derivatives has been investigated in DPPH assay. The result findings of our antioxidant assay are represented in Fig 2. Our result outcomes established that almost all the tested compounds showed excellent antioxidant effect against DPPH Activity. Various concentration ranges of the tested compounds were assayed against DPPH and their IC_50_ values were estimated as well. We concluded that amongst the investigated designed molecules the **C3** was found best potent and showed significant dose dependent antioxidant activity against DPPH screening. The IC_50_ value reported for **C3** was found good as compared to standard drug. Moreover, **C5** and **C6** also showed excellent free radical scavenging effect against DPPH assay. The remaining compounds also showed significant antioxidant effect. Based on the preliminary *in vitro* assays, the highest potent derivatives has been furtherly assayed for *in vivo* as well as histopathological screenings.

### 3.3 Molecular docking studies

The docking analysis was performed to explicate the potential interaction mechanisms of these compounds **C1-C10** *in vitro* with (PDB: 3V99 and 2X08) crystal frameworks. The docking investigation was implemented through Glide’s module. The preliminary inhibitors arachidonic for3V99 and Ascorbic for 2X08 and were redocked into the crystal frameworks to verify the docking methodology. Furthermore, the efficacious performance of the targeted molecules was authenticated *via* the low values of RMSD (2.25 Å (3V99) and 2.01 Å for (2X08), which were acquired through the root mean square deviation between the native and redocked poses of the co-crystallized inhibitors. The binding free energies ΔG were listed in (Table 1). The initial inhibitors have been adequately installed into their binding sites to attain their crystal configurations. The lowest score poses and RMSD revealed increased stability in the binding pocket. The data was utilized to rank the docked poses and to select the most capable docked conformation of each compound. We selected the most perforable docking conformations of all **C1-C10** isoxazole compounds that were detected inside the active site with proper alignment. We noticed that almost all investigated **C1-C10** isoxazole compounds interacted with significant residues in the binding pocket for both enzymes. We defined the inhibitory behavior in the term of binding energy BE for the six **C1-C10** isoxazole that were evaluated with the receptor. Then redocked these compounds, compared the results to the comparative reference inhibitors, and obtained a root mean square deviation (RMSD) which summarized in (Table 1).

**Table 1:** The binding-affinity for compounds C1-C10 with docking scores (kcal/mol).

Compounds	ΔG	rmsd	E._Int_	E._H.B_	E._ele_
**PDB (3V99)**					
**C1**	-5.874	1.493	-40.333	-15.457	-7.605
**C2**	-5.386	1.559	-32.880	-17.102	-7.332
**C3**	-6.096	1.870	-38.683	-13.564	-8.484
**C4**	-6.928	1.102	-17.196	-28.536	-8.688
**C5**	-6.453	1.669	38.520	-19.573	-8.359
**C6**	-6.874	1.869	2.886	-31.697	-8.741
**C7**	-6.661	1.501	36.234	-24.246	-8.753
**C8**	-6.120	1.539	-0.543	-20.820	-8.952
**C9**	-6.038	1.134	-45.754	-25.630	-8.255
**C10**	-5.989	1.611	-34.039	-10.093	-8.927
**Arachidonic**	-6.462	1.258	-75.771	-21.458	-6.872
**PDB (2X08)**					
**C1**	-7.368	0.548	-31.265	-23.534	-12.153
**C2**	-7.505	1.844	-34.649	-19.014	-8.638
**C3**	-7.344	1.228	-31.188	-19.258	-10.809
**C4**	-7.599	1.472	-31.213	-18.328	-9.729
**C5**	-9.706	1.655	18.007	-32.013	-9.552
**C6**	-7.953	0.747	41.606	-25.209	-11.778
**C7**	-8.417	1.662	20.624	-24.987	-9.976
**C8**	-8.417	1.278	51.907	-23.865	-10.032
**C9**	-8.129	1.448	1.967	-29.470	-10.430
**C10**	-8.296	0.885	-33.929	-26.615	-13.638
**Ascorbic**	-7.168	1.206	-37.894	-19.574	-10.759

Where, *ΔG*: Free binding energy of the ligand; RMSD: root-mean-square deviation; *H*.B.: H-bonding energy between protein and ligand; EInt: Binding affinity of H-bond interaction with receptor; Eele: Electrostatic interaction over the receptor.

Using the fingerprint interaction between ligand and protein (PLIF), binding effectiveness was assessed. The "CHARMM " molecular-mechanics force field created the poses, and then picked the pose with the lowest "ΔG" and "RMSD" to evaluate the binding affinities of **C1-C10** *isoxazole* molecules. The glide ΔG score, which calculates the free energy of binding between the ligand and the receptor protein, was used to assess the binding mechanism and stability of the docked compounds. The poses of molecules with the lowest score and RMSD is the more stable in the binding pocket, and docking energy details are listed in Table 1. The interactions between the active **4** molecules and residues of active site were mainly polar bonds, hydrogen bonding, π-π and π-H interactions, which contributed to a strong alignment with the with the enzyme backbone.

#### 3.3.1 Molecular docking of C3 and C5-C9 into LOX-5

Since there is no co-crystal structure of the inhibitor with the human LOX-15 we docked the most active compounds **C3** and **C5-C9** into the human LOX-5 (PDB: 3v99) active site. LOX-5 contributes to the diabetes-induced increase in retinal superoxide generation and inhibitors of this enzyme’s activity have potential application in the prevention and treatment of diabetes retinopathy. Moreover, the crystal structure of LOX-5 with arachidonic acid, combined with both altered substrate and product profiles for the modified enzyme, led to a suggestion that phosphorylation of LOX-5 at Serin-663 converts the enzyme to a robust LOX-15 [37]. Compounds **C3** and **C5-C9** showed healthier interaction efficacy against LOX-5 compared to the reference inhibitor (ΔG = -6.462 kcal/mol), as mentioned in (Table 1). Binding efficiency arranged as; **C6** (ΔG = -6.873 kcal/mol)>**C7** (ΔG = -6.660kcal/mol)>**C5** (ΔG = -6.453kcal/mol)>**C8** (ΔG = -6.119kcal/mol)>**C3** (ΔG = -6.095kcal/mol). Compound **C6** with highest activity against LOX-5 (IC_50_ = 3.67) showed highest binding efficiency compared all investigated compounds **C1-C10** to with ΔG values of -6.873 kcal/mol.

**Fig 4:**
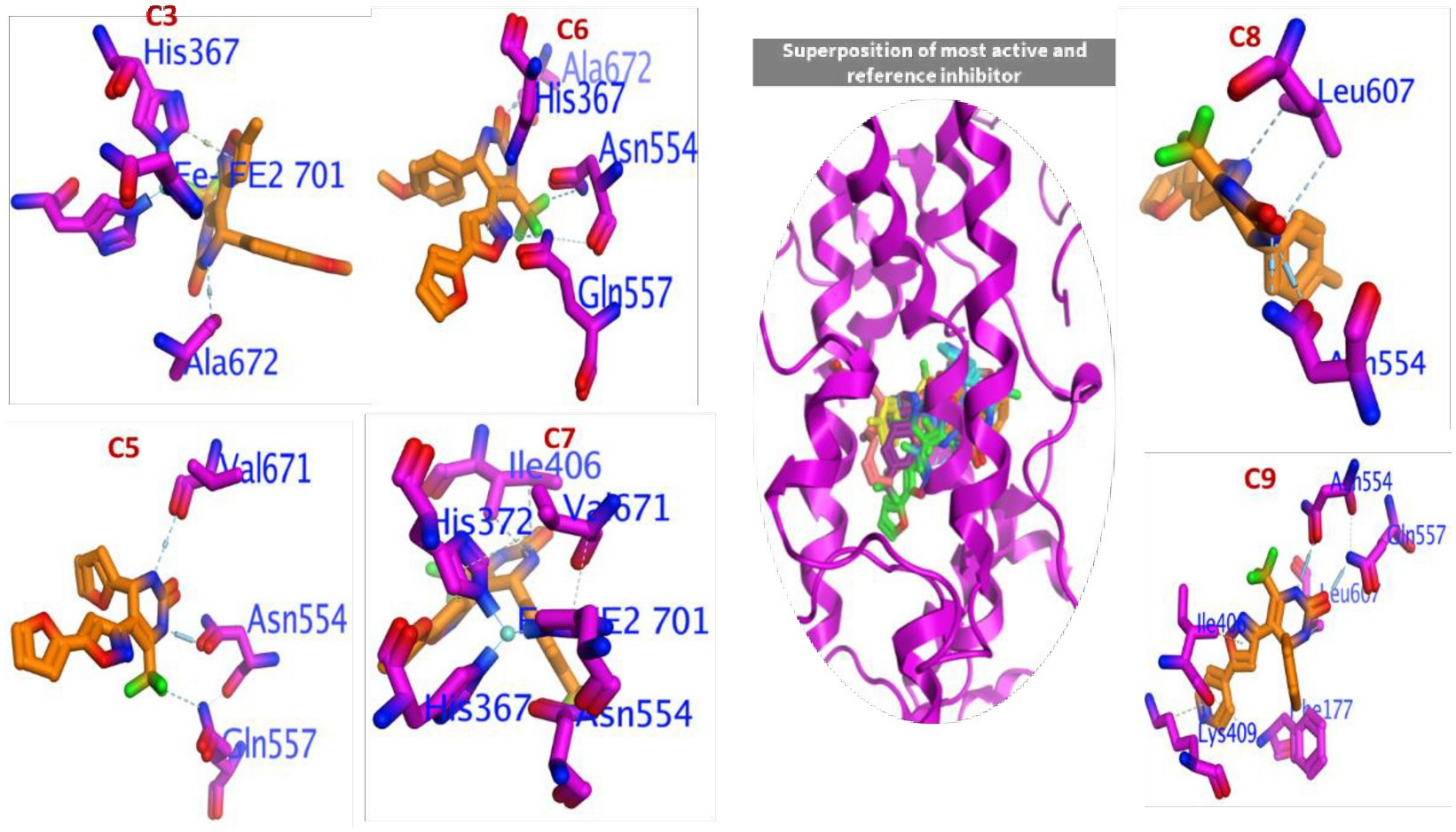
Binding interaction of most active compounds, and superimposed poses of the test compounds and arachidonic acid in the active site of 3v99 to validate the docking protocol.

In addition, Fig 4 showed the most active compounds **C3** and **C5-C9** revealed the following ligand-receptor interactions that are envisaged to stabilize the binding of this compounds in the active pocket of LOX-5: n–π→His367 and n–π→Ala672 (**C3**), π–π→Val671, H→Asn554 and H←Gln557 (**C5**), n–π→Ala672, H→His367, H→Asn554 and H←Gln557 (**C6**), n–π→ile406, n–π→Val671, H→His372, H→His367, and H→Asn554 (**C7**), H→Leu607, and H→Asn554 (**C8**), and n–π→ile406, π–π→Phe177, n–π→Lys409, H→Asn554, H→Gln557, and H→Leu607 (**C9**).

#### 3.3.2. Molecular docking of C1-10 into peroxidase

**Fig 5:**
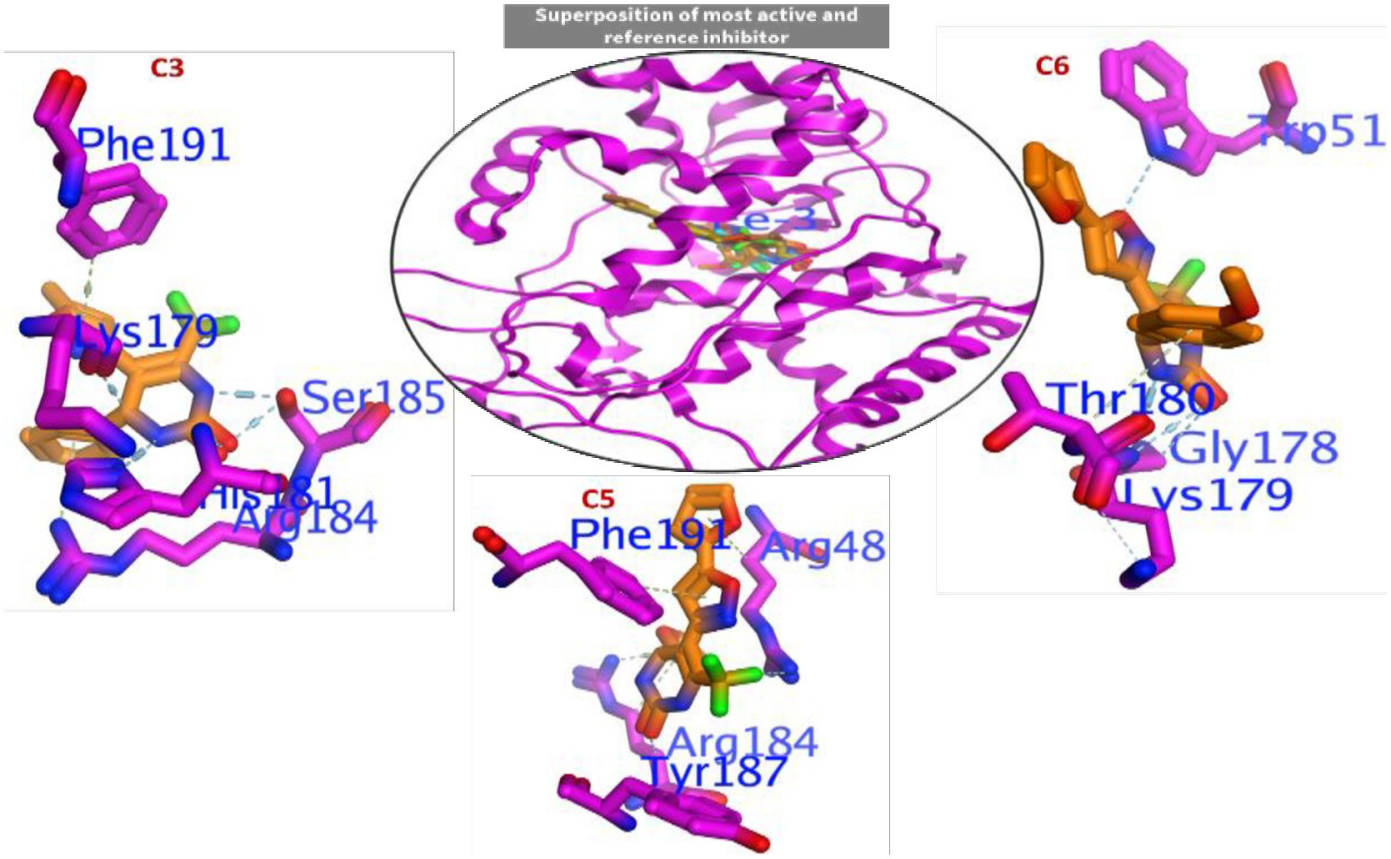
Binding interaction of most active compounds, and superimposed with ascorbic acid as reference inhibitor in active site of 2X08 to validate the docking protocol.

In an attempt to predict the eligibility and validate the activity of the test compounds as potential anti-oxidants at least at theoretical level, we modeled all of them into cytochrome c peroxidase enzyme (PDB code: 2X08) against ascorbic acid as a reference ligand. The isoxazole derivatives are embedded deeply into the binding pocket of cytochrome c peroxidase to interact with vital hydrophilic residues Arg48 and His175 (Fig 5). The binding interactions between all isoxazoles **C1-C10** and the receptor are comparable to that of ascorbic acid (ΔG = -7.168 kcal/mol) with the ΔG values ranging from -7.3 to -9.75 kcal/mol (see Table 1). The *N*-4-methoxyphenyl-phenylisoxazole derivative **C3** and the Furan-2-yl-isoxazoleanalogue **C5** showed higher binding interaction than ascorbic acid with the ΔG values of -7.334 and -9.70 kcal/mol, respectively. The predicted results are consistent with their strong DPPH radical scavenging activity within this series. In addition 5-(5-(furan-2-yl)isoxazol-3-yl)-4-(4-methoxyphenyl analogue in **C6** showed higher binding interaction with ΔG values of -7.383, and -7.952 kcal/mol, respectively. These two compounds exhibited the highest DPPH radical scavenging activity within this category.

**Fig 6:**
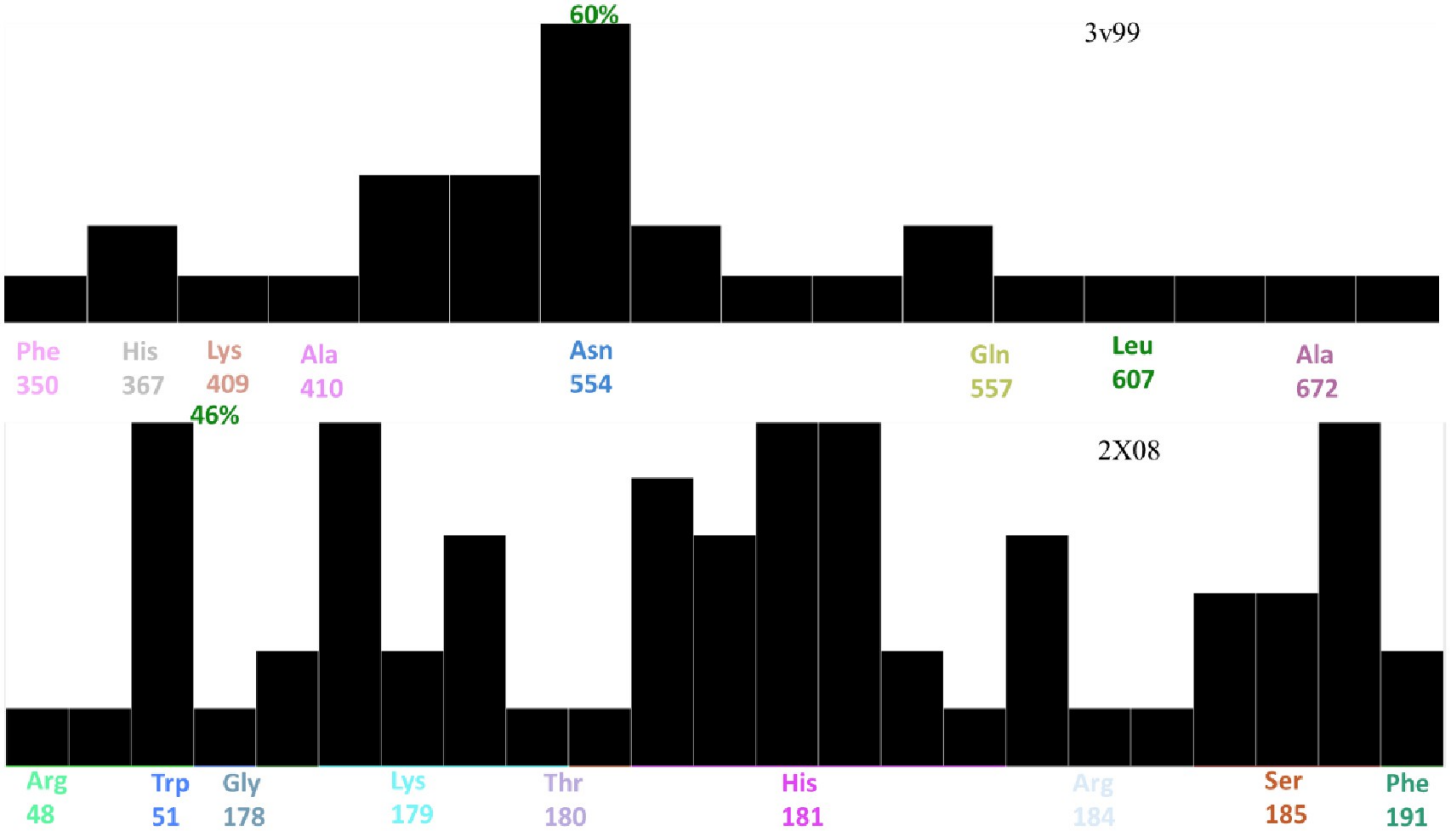
Populations analysis based on PLIF between C1-C10 and ascorbic acid (2X08), and arachidonic acid (3v99).

The binding effectiveness of the inhibitors **C1-C10** was evaluated by the protein-ligand interaction fingerprint (PLIF) method to generate population plot (Fig 6). The PLIF was computed based on the docking results of the inhibitors at the active site of 2X08 and, 3v99 active sites. The Glide (ΔG) score, which calculates the free energy of binding between the ligand and the receptor protein was used to assess the binding mechanism and stability of the docked investigated **C1-C10** compounds. The different types of interactions were classified as hydrogen bonds, ionic and surface interactions according to the remaining components, and created a fingerprint scheme. The population analysis based on PLIF between **C1-C10** and ascorbic acid showed 60% interaction with Asn554 of 2X08, while LOX-5 (3v99) showed 46% interaction score with Arg48 when interacted with arachidonic acid and **C1-C10**.

## 4 Conclusion

The 5-LOX enzymes inhibition analysis has been conducted for the tested derivatives at various concentration strengths. All the compounds were found potent and showed significant percent inhibition of 5-LOX pathway in dose dependent manner. Among the tested compounds, **C6** was found most potent which showed significant 5-LOX percent inhibition assay and also reported the minimum IC_50_ value comparable to the reference drug. The *in vitro* 5-LOX enzymes inhibition assays of **C5** and **C3** also showed non-significant percent inhibition and good potency next to **C6**. Our result outcomes established that almost all the tested compounds showed excellent antioxidant effect against DPPH Activity. Moreover, **C5** and **C6** also showed excellent free radical scavenging effect against DPPH assay. The remaining compounds also showed significant antioxidant effect. Based on the preliminary *in vitro* assays, the highest potent derivatives have been furtherly assayed for *in vivo* as well as histopathological screenings.

## Supporting information

S1 Raw data The supporting information section includes raw data. (XLSX)
